# Dosing Oncology Therapeutics in Combination Therapy for Renal Dysfunction: The University of California San Diego Study of Personalized Cancer Therapy to Determine Response and Toxicity (UCSD-PREDICT) Experience

**DOI:** 10.7759/cureus.3634

**Published:** 2018-11-26

**Authors:** Mina Nikanjam, Jason Wing, Edmund Capparelli, Razelle Kurzrock

**Affiliations:** 1 Oncology, University of California San Diego, La Jolla, USA; 2 Oncology, Oregon Health Sciences University, Portland, USA; 3 Pediatrics, University of California San Diego, La Jolla, USA

**Keywords:** renal dysfunction, targeted therapy, cytotoxic agent, oncology combination therapy

## Abstract

Introduction

Dose reductions are often required to avoid toxicity in combination therapy for advanced cancers, but information on appropriate dose reductions in renal dysfunction is lacking. This study assessed dose reductions of renally cleared oncology agents given in combination therapy in the setting of renal dysfunction.

Methods

A database of 1,072 patients was screened to identify patients with renal dysfunction (glomerular filtration rate < 60 mL/min) receiving oncology combination therapy with at least one agent requiring dose reduction for renal insufficiency. The dose of the renal agent was compared to the single-agent renal dosing recommendations to calculate a dose percentage. Tolerability was determined from electronic medical records review.

Results

Thirty-three regimens (n = 25 patients) were identified: 11 included at least one targeted agent (n = 8 patients) and 22 had only cytotoxic chemotherapy (n = 18 patients). The renal agent was given at the recommended single-agent renal dose in ~50% of combinations; ~50% of all regimens were tolerated, and only six combinations had dose reductions for toxicity. The median final dose percentage was 100% of the recommended renal dose (range: 25% - 333%); no significant differences were seen between groups (cytotoxic - tolerated, cytotoxic - not tolerated, targeted - tolerated, targeted - not tolerated; p = 0.38). No significant differences were observed between tolerated vs. non-tolerated (p = 0.97) or targeted vs. cytotoxic (p = 0.80) regimens.

Conclusions

Dose reductions of renally cleared agents are highly variable in oncology patients with renal dysfunction. Additional studies are needed to determine appropriate dosing adjustments in this population.

## Introduction

Combination treatment approaches have dramatically changed outcomes for numerous advanced malignancies with high cure rates as has been seen in testicular cancer, acute lymphocytic leukemia, and Hodgkin lymphoma [1]. The complicated and heterogeneous molecular and biologic landscapes of metastatic tumors necessitate novel combination therapeutic approaches to improve responses and overcome resistance [2-4]. Thus, novel combinations of cytotoxic chemotherapy and targeted agents, as well as combined targeted therapies, are increasingly being pursued.

Many patients with advanced solid tumors will require dose adjustments for renal dysfunction. Prior studies indicate that 60.3% of patients had an estimated creatinine clearance (CrCl) < 89 mL/min with 16.6% at a CrCl < 60 mL/min (Cockcroft-Gault equation) [5]. Renal dysfunction can occur from co-morbidities prior to cancer diagnosis, can be caused by cancer itself, or can be due to the drugs used to treat cancer [6]. Although an evaluation and study of renal dysfunction dosing are required for many drugs as part of the Food and Drug Administration (FDA) approval, recommendations are limited to single-agent dose-reduction recommendations [7]. Patients with renal dysfunction are generally excluded from combination therapy trials [8]. Thus, the dose reductions required to safely administer renally cleared drugs for combination therapy in the setting of renal dysfunction is unknown.

The aim of the current pilot study was to determine the dose reductions of renally cleared agents given in combination therapy at the University of California San Diego (UCSD) for patients with renal dysfunction, to determine how dosing compared to single-agent recommended renal dose reductions, and to understand how dosing affected the tolerability of the regimens. 

## Materials and methods

This is a retrospective, descriptive, pilot study of patients enrolled in the University of California San Diego Study of Personalized Cancer Therapy to Determine Response and Toxicity (UCSD-PREDICT) [9], which encompasses an institutional review board (IRB)-approved observational cohort study at UCSD designed to learn more about personalized cancer therapy, including dosing, response to treatment, and side effects. This study was performed in accordance with the UCSD IRB guidelines for data analysis and for any investigational treatments for which patients gave consent.

The pilot study evaluated the subset of PREDICT patients whose prior oncology treatment regimens were documented in the PREDICT electronic database. This database had previously been generated from a manual review of the electronic medical records. The database and electronic medical records were accessed to determine prior oncology treatment regimens and renal function at the start of each therapy. Patients were included who had documented renal dysfunction (creatinine clearance by Cockcroft-Gault equation of less than 60 mL/min) and received combination therapy with two or more chemotherapeutic agents where at least one drug required a dose adjustment for the patient’s degree of renal dysfunction. Those who did not receive their full care at UCSD for the entirety of the regimen were excluded as toxicity and dose adjustments were not routinely available from outside records.

Medication administration records, laboratory values, and physician notes were reviewed to determine the dosing throughout the regimen for each drug in the combination and tolerability of the regimen. The starting dose, final dose, and the lowest dose used were documented for all drugs in the regimen. The dose of the drug(s) requiring dose reduction for renal failure in the combination was compared to the recommended single-agent renal dose reduction to determine the dose percentage. Single-agent renal dosing recommendations were determined from package inserts and the clinical pharmacology resource [10]. Tolerability was assessed through a chart review of physician notes. Regimens that required multiple delays in administration (which was verified by physician notes) were discontinued due to toxicity or were explicitly stated by the physician to be poorly tolerated were listed as not tolerated. If a regimen was not tolerated at the starting dose, but side effects and dose delays resolved following dose reduction, the combination was considered to be tolerated. Differences in the initial and final dose percentages based on tolerability and type of regimen (targeted agent included vs. exclusively cytotoxic agents) were compared using an analysis of variance (ANOVA) which was performed in SAS (v. 9.4) (SAS Institute Inc., Cary, NC). Differences between the dosing recommendation category as compared to the renal recommended dose (lower vs. higher than or equal to the recommended renal dose; higher than vs. lower than or equal to the recommended renal dose) were compared to tolerability using Fisher’s exact test in SAS (v. 9.4).

## Results

**Figure 1:**
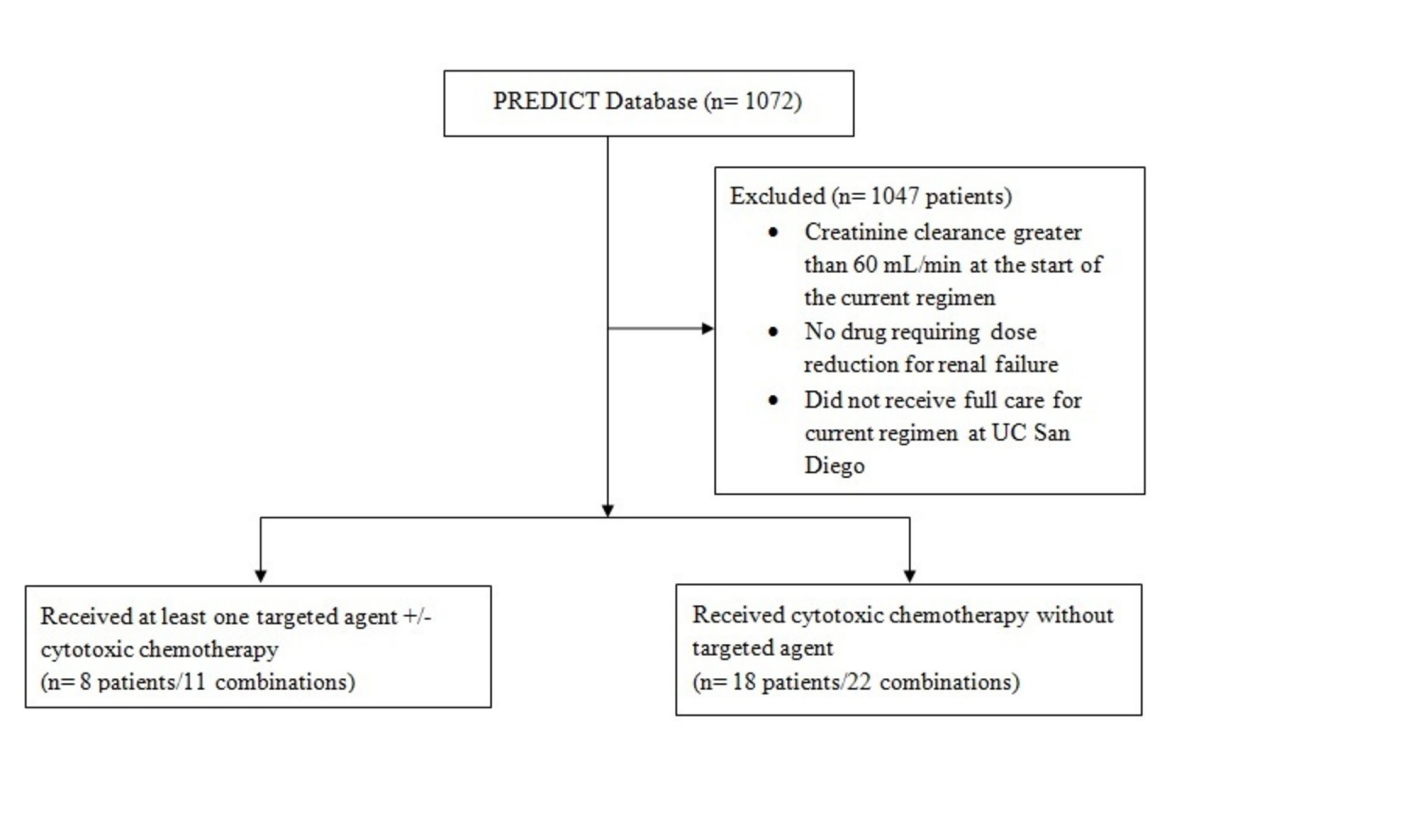
Study Design Consort Diagram Patients were included who had a GFR < 60 mL/min at the start of therapy by the Cockgroft-Gault calculation and were receiving combination therapy with at least one drug that required a dose reduction for renal failure. One patient received a regimen with at least one targeted agent and exclusively cytotoxic chemotherapy, and thus was included in both groups. GFR: glomerular filtration rate; PREDICT: Personalized Cancer Therapy to Determine Response and Toxicity; n: number; UC: University of California

After review of the PREDICT database (n = 1,072 patients), 33 regimens met the inclusion criterion (n = 25 patients). Eleven regimens included at least one targeted agent (n = 8 patients), and 22 regimens were exclusively cytotoxic chemotherapy (n = 18 patients) (Figure 1). One patient was represented in both categories. The most commonly encountered renally cleared drug with a recommendation for dose reduction was carboplatin (n = 24 combinations); no other agent was encountered more than four times. Baseline characteristics at the start of therapy for each patient are shown in Table 1.

**Table 1:** Patient Characteristics at the Start of a Regimen ^a ^Subject with one regimen containing a targeted agent and two cytotoxic regimens was included in both the targeted and cytotoxic therapy grouping for demographics; ^b ^Creatinine clearance calculated by the Cockcroft-Gault formula. CNS: central nervous system; GU: genitourinary; GYN: gynecology; N: number

	At least one targeted therapy (n = 8)	Cytotoxic therapy (n = 18)^ a^
Gender N (%)		
Male	1 (12.5)	7 (38.9)
Female	7 (87.5)	11 (61.1)
Race N (%)		
White	5 (62.5)	10 (55.6)
Black	-	-
Hispanic	-	2 (11.1)
Asian	-	3 (16.7)
Other	3 (37.5)	3 (16.7)
Median Age at Start of Therapy (Range; years)	66.8 (41.4 - 86.2)	70.6 (37 - 85)
Median Number of Drugs in Regimen (Range)	3 (2 - 7)	2 (2 - 3)
Type of cancer N (%)		
Lung	2 (25)	9 (50)
CNS	1 (12.5)	-
Blood	3 (37.5)	1 (5.6)
Skin	1 (12.5)	-
Breast	1 (12.5)	2 (11.1)
GU	-	1 (5.6)
GYN	-	1 (5.6)
Head/Neck	-	3 (16.7)
Liver	-	1 (16.7)
Median creatinine clearance^b^ at the start of therapy (range, mL/min)	40 (29 - 59)	51 (19 - 58)

The chemotherapy regimens, baseline creatinine clearance at the start of each regimen, dose percentages, and tolerability are summarized in Tables 2-3. In only two of the 11 regimens (18%) that included a targeted agent was the renal agent given at the recommended single-agent renal dose, while in 13 of the 22 cytotoxic regimens (59%), the renally cleared agent was administered at the recommended dose. Seven of the 33 total regimens had dose adjustments; six required dose reductions (three had been started at or below the recommended single agent renal dose and three had been started above the single agent renal dose) and one had a dose increase. Three of the 11 targeted regimens (27%) included dose reductions of the renally cleared drug during the course of treatment (two of the three regimens were started at a reduced dose) as compared to three of the 22 cytotoxic regimens (14%) (none of the three regimens were started at a reduced dose). In four of the 11 targeted regimens (36%), a tolerable final dose was found; in 13 of the 22 cytotoxic regimens (59%), a tolerable final dose was found.

**Table 2:** Dosing, Tolerability, and Time-to-treatment Failure for Combination Regimens with At Least One Targeted Therapy ^a ^Patients receiving more than one regimen meeting the inclusion criteria are listed as separate rows in the table; ^b ^CrCl: creatinine clearance based on the Cockcroft-Gault equation; ^c ^Dose percent: (dose administered divided by standard dose recommended for the current renal function) x 100; ^d ^Regimens that were not tolerated had multiple delays in administration, were discontinued due to toxicity, or were explicitly stated by the physician to be poorly tolerated were listed as not tolerated; ^e ^Lowest dose in regimen was 45% but was increased to a final dose of 60%; ^f ^Pemetrexed is contraindicated (CI) at a creatinine clearance of 45 mL/min or less; ​​​​​​​^g ^CyBorD: cyclophosphamide, bortezomib, and dexamethasone; ​​​​​​​^h ^VDR-PACE: bortezomib, lenalidomide, dexamethasone, cisplatin, doxorubicin, cyclophosphamide, and etoposide

ID^a^	Baseline CrCl^b^ (mL/min)	Combination Regimen	Renally Adjusted Medication(s)	Starting Dose Percent^c^	Final Dose Percent^c^	Tolerable (Y/N)^d^
1	29	bevacizumab, carboplatin, gemcitabine	carboplatin	90%	60%^ e^	Y
2	35	bevacizumab, carboplatin, pemetrexed	carboplatin	100%	100%	N
			pemetrexed	CI^f^	CI^f^	
2	55	bevacizumab, cisplatin, pemetrexed	cisplatin	133.30%	133.30%	N
3	57	bevacizumab, carboplatin, erlotinib	carboplatin	83.30%	66.70%	N
4	33	CyBorD^g^	cyclophosphamide	111.10%	111.10%	Y
4	29	VDR-PACE^h^	lenalidomide	333.30%	333.30%	Y
			cisplatin	100%	100%	
			cyclophosphamide	83.30%	83.30%	
			etoposide	100%	100%	
4	40	lenalidomide carfilzomib dexamethasone vorinostat	lenalidomide	250%	125%	N
5	51	CyBorD^g^	cyclophosphamide	111.10%	111.10%	N
6	46	cetuximab carboplatin	carboplatin	100%	100%	Y
7	39	lenalidomide rituximab	lenalidomide	25%	25%	N
8	59	olaparib carboplatin paclitaxel	carboplatin	50%	50%	N
Median	40	-	-	100%	100%	-
(Range)	(29 – 59)			(25% - 333%)	(25% - 333%)	

**Table 3:** Dosing, Tolerability, and Time-to-Treatment Failure for Combination Regimens with Only Cytotoxic Chemotherapy ^a ^Patients receiving more than one regimen meeting the inclusion criteria are listed as separate rows in the table; ^b ^CrCl: creatinine clearance based on the Cockcroft-Gault equation; ^c ^Dose percent: (dose administered divided by standard dose recommended for the current renal function) x 100; ^d ^Regimens that were not tolerated had multiple delays in administration, were discontinued due to toxicity, or were explicitly stated by the physician to be poorly tolerated were listed as not tolerated; ^e ^Gemcitabine may require a dose adjustment at a creatinine clearance less than 30 mL/min; however, no formal recommendation has been provided; ^f ^CLAG-M: cladrabine, cytarabine, G-CSF, and mitoxantrone; ^g ^carboplatin and paclitaxel were administered as two separate treatments during the patient’s disease treatment with different starting CrCL values and were thus considered as two separate regimens for the analysis N: no; Y: yes

ID^a^	Baseline CrCl^b^ (mL/min)	Combination Regimen	Renally Adjusted Medication(s)	Starting Dose Per Cent^c^	Final Dose Per Cent^c^	Tolerable (Y/N)^d^
1	19	carboplatin vinorelbine	carboplatin	50%	50%	N
1	29	carboplatin gemcitabine^e^	carboplatin	60%	60%	Y
9	57	carboplatin gemcitabine paclitaxel	carboplatin	100%	100%	N
9	57	carboplatin gemcitabine	carboplatin	100%	100%	N
10	50	fludarabine cytarabine	fludarabine	200%	200%	N
			cytarabine	166.70%	166.70%	
10	55	CLAG-M^f^	cytarabine	166.70%	166.70%	Y
11	52	carboplatin gemcitabine	carboplatin	100%	100%	N
12^g^	47	carboplatin paclitaxel	carboplatin	83.30%	83.30%	N
12^g^	54	carboplatin paclitaxel	carboplatin	83.30%	83.30%	Y
13	46	carboplatin gemcitabine	carboplatin	120%	100%	N
14	52	carboplatin pemetrexed	carboplatin	80%	100%	Y
15	44	carboplatin paclitaxel	carboplatin	120%	80%	N
16	53	carboplatin pemetrexed	carboplatin	100%	100%	Y
17	48	carboplatin gemcitabine	carboplatin	120%	100%	N
18	51	carboplatin paclitaxel	carboplatin	100%	100%	Y
19	58	carboplatin etoposide	carboplatin	100%	100%	Y
20	44	carboplatin gemcitabine	carboplatin	100%	100%	Y
21	51	cyclophosphamide	cyclophosphamide	111.10%	111.10%	Y
		doxorubicin				
22	43	carboplatin pemetrexed	carboplatin	100%	100%	Y
23	48	carboplatin etoposide	carboplatin etoposide	100%	80%	Y
				100%	66.70%	
24	55	carboplatin pemetrexed	carboplatin	100%	100%	Y
25	58	carboplatin docetaxel	carboplatin	100%	100%	Y
Median	51	-	-	100%	100%	-
(Range)	(19 – 58)			(50% - 200%)	(50% - 200%)	

**Figure 2:**
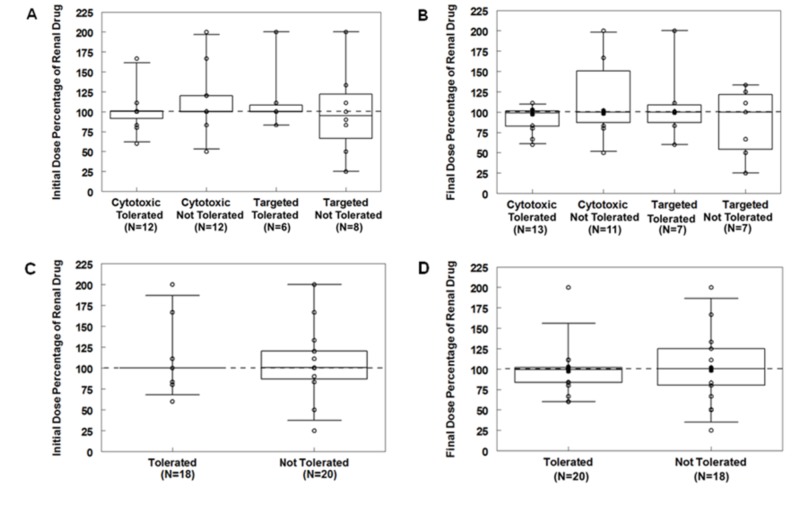
Dose Percentages of Drugs Requiring Dose Reductions for Renal Failure The type of regimen was compared to A) initial (p = 0.57) and B) final dose percentages (p = 0.38), while tolerability was compared to C) initial (p = 0.89) and D) final dose percentages (p = 0.97) with no significant differences seen between the groups. Regimens containing more than one renally cleared drug are represented as separate points on the graph. Dose percentage: (dose administered divided by standard dose recommended for the current renal function) x 100. Regimens that were not tolerated either had multiple delays in administration, were discontinued due to toxicity, or were explicitly stated by the physician to be poorly tolerated. Dose percentages above 200 were assigned a value of 200 (n = 1) for the graph. No significant differences were seen between the groups.

A graphical representation of the dose percentages (dose administered divided by recommended single-agent renal dose) for the renal drugs in the combinations for the initial dose is shown in Figures 2A, 2C and for the final dose in Figures 2B, 2D. For the initial dose, the median dose percentage was 100% for the cytotoxic - tolerated (n = 12 drugs), cytotoxic - not tolerated (n = 12 drugs), and the targeted - tolerated (n = 6 drugs) groups, and 95% for the targeted - not tolerated (n = 8 drugs) groups with no difference seen between the groups. For the final dose, the median dose percentage was 100% for each group (cytotoxic - tolerated (n = 13 drugs), cytotoxic - not tolerated (n = 11 drugs), targeted - tolerated (n = 7 drugs), and targeted - not tolerated (n = 7 drugs)) and no significant differences were seen between the groups. There were also no significant differences in dose percentage given observed between regimens that were tolerated (initial dose: n = 18, final dose: n = 20 drugs) versus not tolerated (initial dose: n = 20 drugs, final dose: n = 18 drugs) and targeted (n = 14 drugs) versus cytotoxic (n = 24 drugs) regimens (initial dose: p = 0.49, final dose: p = 0.80). Tolerability was not found to be affected by dosing higher than recommended or lower than recommended (Table 4). Thus, tolerability was not found to be related to or affected by the dose of the renal agent, although this may be secondary to the small sample size of the pilot study.

**Table 4:** Dosing Versus Tolerability and Outcome ^a ^Regimens that were not tolerated had multiple delays in administration, were discontinued due to toxicity, or were explicitly stated by the physician to be poorly tolerated were listed as not tolerated; ^b ^Dose percentage (dose administered divided by standard dose recommended for the current renal function) x 100 of 100% were at the recommended renal dose, those greater than 100% were higher than the recommended renal dose, and those less than 100% were less than the recommended renal dose; ^c ^Three subjects had more than one drug requiring dose reduction for renal dysfunction in the combination N: number

	Tolerated^a^	Not tolerated^a^	Tolerated p-value
Initial dosing versus tolerability and outcome			
Lower than/equal to the recommended dose (N = 26 drugs) ^b, c^	14	12	0.31
Higher than the recommended dose (N = 12 drugs)	4	8	
Lower than the recommended dose (N = 10 drugs)	4	6	0.72
Higher than/equal to the recommended dose (N = 28 drugs)	14	14	
Final dosing versus tolerability and outcome			
Lower than/equal to the recommended dose (N = 29 drugs)	17	12	0.7
Higher than the recommended dose (N = 9 drugs)	4	5	
Lower than the recommended dose (N = 12 drugs)	6	6	0.73
Higher than/equal to the recommended dose (N = 26 drugs)	11	15	

## Discussion

Combination therapy in oncology is essential to improve outcomes and limit resistance. However, dosing of combination therapy can be challenging. The current trend of dosing combination therapy is a restrictive approach, which focuses on strategic dosing and drug administration in order to spare normal cells, while simultaneously creating a targeted cytotoxic effect on cancer cells [11]. Recent studies have demonstrated that the tolerability of drugs may change when given in combination, necessitating dose reductions for novel combinations [12-15]. However, these studies did not address organ dysfunction nor inform about appropriate dosing reductions for combination therapy. Many oncology therapeutics undergo renal clearance and moderate to severe renal dysfunction often occurs in patients with cancer. Thus, understanding how to administer anti-cancer therapies in the setting of organ dysfunction is important to provide safe and efficacious therapies.

The current pilot study aimed to determine dosing and tolerability for oncology patients with renal dysfunction receiving combination therapy in a university-based practice environment. It focused on renally cleared agents given in combination therapy to patients with moderate and severe renal dysfunction and determined how dose reductions compared to standard single-agent renal dose reduction recommendations, as well as how well the combinations were tolerated. The renal agent was given at the recommended single-agent dose reduction for renal failure in only slightly less than half of the combinations. No significant differences were found in the initial or final dose percentages administered between exclusively cytotoxic regimens versus those including targeted agents or with tolerability of the regimen. The majority of the exclusively cytotoxic regimens were administered at the single-agent recommended renal dose, and these regimens were better tolerated than regimens with targeted agents. This is likely due to the fact that fewer drugs were in the cytotoxic regimens (median: two vs. three drugs) and many of these regimens contained carboplatin, which is dosed based on renal function. Interestingly, despite the fact that many of the regimens were poorly tolerated (defined by delays in administration, discontinuation due to toxicity, or comments in physician notes), dose reductions of the renal agent were not routinely made to improve tolerability in the majority of cases. The regimens were discontinued or drugs were removed rather than attempting a dose reduction. Dose reduction of the non-renal agent was also uncommon among regimens that were not tolerated.

Carboplatin was the most commonly used renally cleared drug, and the dose was personalized for renal function (n = 24 combinations). Three regimens started at more than 100% of the dose and all were dose-reduced for toxicity, while one regimen started at 80% of the renal dose with a subsequent increase to the full renal dose. Lower doses were given due to other co-morbidities in some cases. The final doses ranged from 50% - 100% of the renal recommended dose with nine of the 24 regimens dosing carboplatin at less than 100% of the single-agent renal recommended dose.  

Renal dysfunction justifying dose modification occurs frequently in patients with cancer. The majority of renal dose reductions occur in moderate to severe renal dysfunction (glomerular filtration rate (GFR) < 60 mL/min/1.73 m^2^) [10]. The first and second Renal Insufficiency and Cancer Medications studies (IRMA-1 and IRMA-2) were cohort studies of close to 5,000 patients each which looked at the prevalence of renal insufficiency in oncology patients and found 16.6% and 11.9% of patients had GFR values in this range, respectively [5, 16]. Other studies have demonstrated GFR levels < 60 mL/min/1.73 m^2^ of 22.0% [17], 16.1% [18], and 14.7% [19]. Given the prevalence of renal dysfunction in the oncology patient population, it was surprising that so few combinations were found in the UCSD PREDICT database. This may reflect the avoidance of drugs with a renal clearance in this population.

The IRMA-2 study also demonstrated a reduced survival for cancer patients with renal dysfunction (16.4 vs. 25.0 months) and higher rates of dose-related adverse events [20]. Chen et al. found that 35% of colorectal cancer patients with normal creatinine values had a GFR less than 60 mL/hr [21], which led to a longer toxicity duration and the development of significantly more Grade 1 and 2 toxicities. In contrast, a study by Lichtman et al. of elderly patients with early breast cancer receiving either cyclophosphamide/doxorubicin (AC) or cyclophosphamide/methotrexate/fluorouracil (CMF) found no significant effect on relapse-free or overall survival with lower calculated GFRs [22].

The current pilot study is limited by a small sample size. Thus. the results may not be generalizable and were too small to accurately assess outcome differences with dosing. The majority of regimens had carboplatin as the renally cleared drug, but the dose adjustments of other drugs may be different. Due to the retrospective nature of the study, it was difficult to determine why the renal dose reductions differed from single-agent recommendations, and the use of these drugs in renal dysfunction may be limited as physicians opted to use alternative therapies and regimens. 

## Conclusions

In conclusion, the current pilot study demonstrated significant variability in dose adjustments for renally cleared drugs given in combination therapy in the presence of renal dysfunction, even for carboplatin. Toxicity was seen particularly in combinations with targeted agents. The regimen or a drug in the combination was discontinued to address toxicity rather than attempting a dose reduction in the majority of cases. Additional studies are needed to determine the appropriate dosing adjustments for renally cleared agents given in combination therapy to patients with renal dysfunction.

## References

[1] DeVita VT Jr, Chu E (2008). A history of cancer chemotherapy. Cancer Res.

[2] Kurzrock R, Giles FJ (2015). Precision oncology for patients with advanced cancer: the challenges of malignant snowflakes. Cell Cycle.

[3] Schwaederle M, Chattopadhyay R, Kato S (2017). Genomic alterations in circulating tumor DNA from diverse cancer patients identified by next-generation sequencing. Cancer Res.

[4] Kato S, Krishnamurthy N, Banks KC (2017). Utility of genomic analysis in circulating tumor DNA from patients with carcinoma of unknown primary. Cancer Res.

[5] Launay-Vacher V, Oudard S, Janus N (2007). Prevalence of renal insufficiency in cancer patients and implications for anticancer drug management: the Renal Insufficiency and Anticancer Medications (IRMA) study. Cancer.

[6] Superfin D, Iannucci AA, Davies AM (2007). Commentary: Oncologic drugs in patients with organ dysfunction: a summary. Oncologist.

[7] (2018). Guidance for industry: pharmacokinetics in patients with impaired renal function — study design, data analysis, and impact on dosing and labeling. http://www.fda.gov/downloads/drugs/guidances/ucm204959.pdf..

[8] Malik L, Mejia A, Weitman S (2014). Eligibility of patients with renal impairment for phase I trials: time for a rethink?. Eur J Cancer.

[9] UCSD PREDICT (2018). Study of Personalized Cancer Therapy to Determine Response and Toxicity (UCSD_PREDICT). http://clinicaltrials.gov/ct2/show/NCT02478931..

[10] (2018). Clinical pharmacology. http://www.clinicalpharmacology.com/..

[11] Bayat Mokhtari R, Homayouni TS, Baluch N, Morgatskaya E, Kumar S, Das B, Yeger H (2017). Combination therapy in combating cancer. Oncotarget.

[12] Nikanjam M, Liu S, Kurzrock R (2016). Dosing targeted and cytotoxic two-drug combinations: lessons learned from analysis of 24,326 patients reported 2010 through 2013. Int J Cancer.

[13] Liu S, Nikanjam M, Kurzrock R (2016). Dosing de novo combinations of two targeted drugs: towards a customized precision medicine approach to advanced cancers. Oncotarget.

[14] Nikanjam M, Liu S, Yang J, Kurzrock R (2017). Dosing three-drug combinations that include targeted anti-cancer agents: analysis of 37,763 patients. Oncologist.

[15] Nikanjam M, Patel H, Kurzrock R (2017). Dosing immunotherapy combinations: analysis of 3,526 patients for toxicity and response patterns. Oncoimmunology.

[16] Janus N, Oudard S, Beuzeboc P (2009). Prevalence of renal insufficiency in cancer patients: data from the IRMA-2 study. J Clin Oncol.

[17] Canter D, Kutikov A, Sirohi M (2011). Prevalence of baseline chronic kidney disease in patients presenting with solid renal tumors. Urology.

[18] Janus N, Launay-Vacher V, Byloos E (2010). Cancer and renal insufficiency results of the BIRMA study. Br J Cancer.

[19] Königsbrügge O, Lötsch F, Zielinski C, Pabinger I, Ay C (2014). Chronic kidney disease in patients with cancer and its association with occurrence of venous thromboembolism and mortality. Thromb Res.

[20] Launay-Vacher V, Janus N, Spano J (2009). Impact of renal insufficiency on cancer survival: results of the IRMA-2 study. J Clin Oncol.

[21] Chen J, Wang XT, Luo PH, He QJ (2015). Effects of unidentified renal insufficiency on the safety and efficacy of chemotherapy for metastatic colorectal cancer patients: a prospective, observational study. Support Care Cancer.

[22] Lichtman SM, Cirrincione CT, Hurria A (2016). Effect of pretreatment renal function on treatment and clinical outcomes in the adjuvant treatment of older women with breast cancer: Alliance A171201, an ancillary study of CALGB/CTSU 49907. J Clin Oncol.

